# Process Optimization of Spray-Dried Aquafaba and Comparison with Freeze-Drying: Techno-Functional Performance and Structural Attributes

**DOI:** 10.3390/foods15162848

**Published:** 2026-08-15

**Authors:** Merve Tuğçe Tunç Odabaş, Furkan Türker Sarıcaoğlu, Mahmut Ekrem Parlak, Arda Akdoğan, Halil İbrahim Odabaş, Engin Gündoğdu, Senay Simsek, İlyas Atalar

**Affiliations:** 1 Faculty of Engineering and Natural Sciences, Department of Food Engineering, Gümüşhane University, Gümüşhane 29100, Turkey; 2 Faculty of Engineering and Natural Sciences, Department of Food Engineering, Bursa Technical University, Bursa 16310, Turkey; furkan.saricaoglu@btu.edu.tr (F.T.S.);; 3 Faculty of Agriculture, Department of Food Engineering, Eskişehir Osmangazi University, Eskişehir 26040, Turkey; 4 Whistler Center for Carbohydrate Research, Department of Food Science, Purdue University, West Lafayette, IN 47907, USA

**Keywords:** aquafaba, spray-drying, optimization, freeze-drying, techno-functional properties

## Abstract

This study aimed to optimize the spray-drying (SD) process for aquafaba and compare the physical and techno-functional properties of the resulting powder with those of its freeze-dried (FD) counterpart. A Box–Behnken design was employed to evaluate the effects of inlet air temperature (150–190 °C), air speed (3.5–4.3 m/s), and feed flow rate (0.3–0.5 L/h) on 14 quality responses. The optimized SD conditions were determined to be an inlet air temperature of 189 °C, an air speed of 4.2 m/s, and a feed flow rate of 0.3 L/h. Validation experiments demonstrated that the developed models had high predictive capacity, with only a small discrepancy (0.56–5.88%) between the predicted and experimental values. Comparative analysis showed that the optimized SD powder had significantly lower moisture content (2.47%) and water activity (0.18) than the FD powder (3.51% and 0.34, respectively), indicating superior storage stability. In addition, the SD powder exhibited greater whiteness (82.37), higher water solubility (88.44%), and substantially greater foaming capacity (266.67%) than the FD sample (243.33%). Although the FD powder demonstrated better wettability and water absorption capacity because of its porous structure, FTIR spectroscopy and protein secondary structure analysis confirmed that SD preserved the functional integrity of aquafaba. Specifically, SD induced a transition from disordered random-coil structures to more ordered β-sheet and β-turn configurations, thereby improving foaming performance. Overall, these findings indicate that optimized spray-drying is a highly efficient and industrially scalable alternative to freeze-drying for producing functional aquafaba powder for use as a plant-based egg substitute.

## 1. Introduction

Aquafaba, the viscous liquid obtained by pressure-cooking legumes such as chickpeas, has emerged as a promising functional ingredient in food technology due to its unique physicochemical and techno-functional properties [1]. As a cost-effective, plant-based alternative to eggs, aquafaba has gained considerable attention for its application in gluten-free, vegan, and reduced-calorie food formulations [2]. Its excellent foaming, emulsifying, gelling, and thickening properties enable its use in a wide range of food systems, including bakery products, confectionery, emulsified foods, and emerging applications such as 3D food printing [3]. In addition to its functionality, aquafaba is a sustainable ingredient that valorizes a by-product of legume processing, thereby contributing to waste reduction and the development of circular food systems [1,3]. However, the high moisture content and inherent instability of liquid aquafaba limit its commercial distribution, storage stability, and direct incorporation into dry food formulations [4]. Consequently, converting aquafaba into a stable powder through drying technologies offers an effective strategy to improve shelf life, facilitate transportation and storage, and expand its industrial applicability.

Several drying techniques have been investigated for producing aquafaba powder, including vacuum drying, oven drying, foam-mat drying, freeze-drying, and spray-drying [4,5]. Among these methods, spray-drying has attracted particular interest because of its high drying efficiency, continuous operation, relatively low processing cost, and widespread industrial application for producing stable powders with desirable functional properties [6]. Compared with freeze-drying, spray-drying provides several practical advantages, including substantially lower production costs, shorter processing times, lower energy requirements, and greater scalability for commercial manufacturing. Nevertheless, the quality and functionality of spray-dried powders depend heavily on both the characteristics of the feed material and the optimization of drying conditions. In particular, processing variables such as inlet air temperature, air speed, and feed flow rate strongly influence moisture removal, powder yield, particle morphology, density, flowability, solubility, and key techno-functional properties, including foaming and emulsifying capacities [7,8]. These processing-induced structural modifications can ultimately determine the suitability of aquafaba powder for specific food applications.

Although previous studies have demonstrated that both spray-drying and freeze-drying can preserve the functional characteristics of aquafaba [2], existing research has primarily focused on either general functionality or selected physical properties of the resulting powders. For example, Begliyev, Yavuz [9] primarily evaluated the physical characteristics of spray-dried aquafaba. In contrast, a comprehensive optimization of spray-drying parameters and their combined effects on both physical and techno-functional properties remains limited. Furthermore, few studies have systematically compared optimized spray-dried powders with freeze-dried powder while simultaneously evaluating molecular, structural, and functional changes associated with the drying process. A more comprehensive understanding of these relationships is essential for maximizing product functionality and facilitating industrial adoption.

Therefore, this study aimed to bridge this knowledge gap by systematically investigating the influence of spray-drying process parameters on the physical, structural, and techno-functional properties of aquafaba powder. Specifically, the objectives of this study were to: (1) evaluate the effects of inlet air temperature, air speed, and feed flow rate on the physical and techno-functional properties of aquafaba powder using Response Surface Methodology (RSM); (2) identify the optimum spray-drying conditions for producing high-quality aquafaba powder; (3) elucidate how thermal atomization during spray-drying induces controlled conformational rearrangements and (4) compare the optimized spray-dried powder with its freeze-dried counterpart to assess their relative functional performance and industrial potential. Unlike previous studies that examined isolated physical parameters or general functionality, this study simultaneously models and optimizes 14 distinct physicochemical, physical, and techno-functional quality responses using a systematic Response Surface Methodology (RSM) framework. This integrated approach provides valuable insight into process optimization while supporting the commercial production of functional aquafaba powder as a sustainable plant-based egg alternative.

## 2. Materials and Methods

### 2.1. Preparation of Aquafaba for Spray-Drying Trials

Dried chickpeas (*Koçbaşı cultivar*) obtained from a local store (Demmar Market, Gumushane, Türkiye), were washed and soaked in tap water at 40 °C for 2 h with a 1:4 (*w*/*v*) ratio. The chickpeas were then pressure-cooked at 0.1 MPa for 30 min at a 1:2 chickpea: water ratio. After separation using a 0.25 mm stainless steel sieve, the initial total solids content was determined to be 6.17 ± 0.25 g/100 mL. The aquafaba was concentrated using a rotary evaporator (Hei-VAP, Heidolph, Bavaria, Germany) at 0.03 MPa until a solid content of 15 g/100 mL was reached.

#### 2.1.1. Spray-Drying

Samples (200 mL) were homogenized at 10,000 rpm (SilentCrusher M, Heidolph, Germany) and dried using a laboratory-scale spray-drier (SD 06, LabPlant, Filey, UK). Independent variables for response surface optimization included drying temperature (150–190 °C), air flow rate (3.5–4.3 m/s), and feed flow rate (0.3–0.5 L/h), as determined by preliminary studies and the physical behavior of the aquafaba matrix during drying. A lower temperature than those in the tested range would lead to insufficient evaporation, resulting in high powder moisture content and severe wall deposition (stickiness) inside the drying chamber. Conversely, higher temperatures would cause excessive thermal denaturation of soluble proteins, degradation of thermolabile compounds, and surface crusting of the particles. Similarly, the feed flow rate and air speed ranges were calibrated to ensure optimal residence time and droplet atomization; higher feed rates led to incomplete drying and nozzle clogging, whereas lower feed rates caused heat damage from prolonged exposure to high-temperature air. Atomization pressure (2 bar) and nozzle diameter (0.5 mm) remained constant in all experiments.

#### 2.1.2. Freeze-Drying

Concentrated aquafaba was frozen at −40 °C for 24 h and dried in a freeze-dryer (LGJ-121, Beijing Fourth Ring Scientific Instrument Co., Ltd., Beijing, China) for 48 h. The resulting product was ground using a laboratory mill (M20, IKA) to produce a fine powder. The freeze-dried cake was gently ground under controlled conditions and passed through a standardized sieve (212 µm) to obtain a uniform particle-size fraction prior to analytical testing.

### 2.2. Physical Properties

#### 2.2.1. Powder Yield

Powder yield was calculated as the ratio of the total solid content in the resulting powder to the total solid content in the feed solution (Equation (1))

(1) $$Powder\;yield\left(\%\right)=\frac{Powder\;weight(g)\times Powder\;dry\;matter\;content(\%)}{Total\;solid\;content\;in\;the\;feed\;solution(g)}$$

#### 2.2.2. Moisture and Water Activity

Moisture was measured at 100 °C using an infrared moisture analyzer (MB 45, Ohaus, Greifensee, Switzerland), and water activity was determined with a water activity meter (Novasina, Lachen, Switzerland).

#### 2.2.3. Powder Density

Powder density was calculated by dividing the powder weight by its volume in a 10 mL graduated cylinder. Results were expressed as kg powder/m^3^ [10].

#### 2.2.4. Angle of Repose (AOR)

The angle of repose was determined using the fixed funnel method [11]. The angle of repose (θ) was calculated using the following formula (Equation (2)).

(2) θ=arctan(h/r)

where

θ = Angle of repose (°)
h = Height of the powder cone (mm)
r = Radius of the base of the cone (mm)

#### 2.2.5. Hygroscopicity

The hygroscopicity of the aquafaba powder samples was determined by storing 0.25 g of powder in a desiccator with saturated NaCl at 25 °C for one week. The hygroscopicity was calculated with the following equation (Equation (3))

(3) $$\mathrm{Hygroscopicity}\;\left(g/100g\right)=\frac{\Delta m/(M+Mi)}{1+(\frac{\Delta m}{M})}$$

where Δm (g) is the increase in weight of aquafaba powder after equilibrium, M is the initial mass of aquafaba powder (g), and Mi is the free water content of the aquafaba powder before exposure to the humid air environment (g/100 g).

#### 2.2.6. Whiteness

Color values of aquafaba powder samples were measured using a Colorimeter (CR-300, Konica-Minolta, Osaka, Japan), which measures the color of an object in terms of L*a*b* values. Whiteness value was calculated from these values using the following equation (Equation (4))

(4) $$Whiteness=100-{\left[{\left(100-L\right)}^{2}+a^{2}+b^{2}\right]}^{\frac{1}{2}}$$

### 2.3. Techno-Functional Properties

#### 2.3.1. Wettability

Wettability was measured as the time (in seconds) required for 0.1 g of powder to become completely wet on the surface of 100 mL of distilled water at 25 °C [12].

#### 2.3.2. Water Solubility Index and Water Absorption Capacity

The water solubility index (WSI) of the aquafaba powder samples was determined as described by Jafari, Ghalegi Ghalenoei [13], with minor modifications. Briefly, 0.5 g of aquafaba powder was suspended in 10 mL of distilled water in a pre-weighed 15 mL centrifuge tube. The suspension was vortexed for 1 min and then incubated in a water bath at 37 °C for 30 min. The mixture was centrifuged at 4 °C and 3500 rpm for 20 min. The supernatant was transferred to a pre-weighed glass Petri dish and dried at 105 °C in a laboratory oven until a constant weight was reached. The centrifuge tube containing the remaining pellet was then reweighed. The water solubility index was calculated using the following equation (Equation (5)).

(5) $$Water\;solubility\;index\left(\%\right)=\frac{Weight\;of\;dried\;supernatant\;(g)}{Total\;solid\;content\;in\;the\;aquafaba\;powder\;(g)}\times 100$$

Water absorption capacity (WAC) was determined after incubating a 0.5 g/10 mL suspension at 37 °C for 30 min, followed by centrifugation at 3500 rpm. WAC was calculated using the following equation (Equation (6)).

(6) $$Water\;absorption\;capacity\left(g/g\right)=\frac{Weight\;of\;residue\left(g\right)-inital\;weight\;of\;sample(g)}{inital\;weight\;of\;powder\;(g)}$$

#### 2.3.3. Oil Absorption Capacity (OAC)

Oil absorption capacity of the aquafaba powder samples was determined by suspending 0.25 g of powder in 5 mL of sunflower oil, vortexing for 30 s, and centrifuging at 3500 rpm after a 30 min incubation period (Equation (7)) [14].

(7) $$Oil\;absorption\;capacity\left(g/g\right)=\frac{Weight\;of\;residue\left(g\right)-inital\;weight\;of\;sample(g)}{inital\;weight\;of\;powder\;(g)}$$

#### 2.3.4. Foaming Capacity (FC)

A 0.2 g powder sample was hydrated, homogenized at 12,000 rpm, and evaluated based on foam volume ($V_{f}$) relative to the initial liquid volume ($V_{i}$) using the following equation (Equation (8)) [5].

(8) $$Foaming\;capacity\;(\%)=\frac{V_{f}{-V}_{i}}{V_{f}}\times 100$$

#### 2.3.5. Emulsion Activity Index

The Emulsion Activity Index (EAI) was determined according to the method of Pearce and Kinsella [15] with slight modifications. An emulsion was prepared by mixing 50 mL of a 10 g/L protein solution with 2 mL of corn oil (yielding a water-to-oil ratio of 25:1). The mixture was homogenized using a high-shear homogenizer (Ultra-turrax T25, IKA-Werke GmbH & Co. KG, Staufen, Germany) at 20,000 rpm for 1 min. Immediately following homogenization (0 min), a 50 μL aliquot was aspirated from the bottom of the tube and diluted into 5 mL of a 1 g/L sodium dodecyl sulfate (SDS) solution. The absorbance of the diluted emulsion was measured at 500 nm using a UV–visible spectrophotometer. The EAI (m^2^/g) represents the interfacial area stabilized per unit protein weight (Equation (9)).

EAI = (4.606 × A0 × N)/(Φ × C × 10,000)
(9)

where A0 is the absorbance of the diluted emulsion at 0 min, N is the dilution factor (×1000), C is the weight of protein per volume of aqueous phase (g/mL), and Φ is the oil fraction of the emulsion.

#### 2.3.6. Soluble Protein Content

The soluble protein content was determined via the Bradford method [16]. Briefly, 0.1 g of powder was solubilized in 10 mL of distilled water for 1 h, centrifuged, and the supernatant was analyzed using bovine serum albumin as a standard $\left(Y=0.3933x+0.0893,\;R^{2}=0.995\right).$

### 2.4. Morphological and Structural Properties

#### 2.4.1. FTIR Spectra

The functional groups and secondary structural changes in the aquafaba powders were analyzed using an FTIR spectrometer (Bruker Optics GmbH, Ettlingen, Germany). Spectra were recorded in the wavenumber range of 4000–400 cm^−1^ at a resolution of 4 cm^−1^ to identify characteristic bands. All measurements were performed in triplicate using a diamond triple-bounce ATR accessory Secondary structures (α-helix, β-sheet, β-turn, and random coil) were identified from the amide I region (1600–1700 cm^−1^). Peakfit 4.12 software was used to calculate the secondary structure content of the powders. The assignment of each sub-peak to a secondary structure was as follows: α-helix, 1648–1664 cm^−1^; β-sheet, 1615–1637 cm^−1^ and 1682–1700 cm^−1^; β-turn, 1664–1681 cm^−1^; and random coil, 1637–1648 cm^−1^ [17].

#### 2.4.2. Surface Morphology

The surface morphologies of the aquafaba powders were examined using scanning electron microscopy (SEM, JSM-7001F, Jeol, Tokyo, Japan). The samples were sputter-coated with a 10 nm gold-palladium layer (Quorum SC7620, Laughton, UK) prior to imaging. Observations were performed at 500×, 1000×, and 2000× magnifications.

#### 2.4.3. Statistical Analysis

The experimental data were reported as mean values accompanied by their respective standard deviations. ANOVA was used to assess statistical significance, and the optimal predictive models were developed using multiple linear regression. This modeling involved evaluating linear, quadratic, and cubic components, along with their interactions, using Design-Expert software (Version 13.0, Stat-Ease Inc., USA). All statistical evaluations were performed at a 95% confidence level. To validate the models, experimental and predicted values were compared using the independent *t*-test. The results demonstrated consistency within the 95% prediction intervals.

## 3. Results and Discussion

### 3.1. Model Fitting

The experimental results obtained under the different spray-drying conditions are presented in Table 1. The adequacy and predictive performance of the response surface models were evaluated using several statistical indicators, including the coefficient of determination (R^2^), adjusted coefficient of determination (Adj. R^2^), predicted coefficient of determination (Pred. R^2^), coefficient of variation (CV), and adequate precision (Ad. Prec.) (Table 2). Overall, the developed models demonstrated excellent goodness-of-fit and predictive capability, indicating that the selected processing variables, inlet air temperature, air speed, and feed flow rate, were appropriate for describing the physical and techno-functional responses of aquafaba powder.

The R^2^ values exceeded 0.96 for most responses, indicating that the regression models explained more than 96% of the variability in the experimental data. Although comparatively lower R^2^ values were observed for water absorption capacity (0.85), soluble protein content (0.89), and oil absorption capacity (0.72), these values remain acceptable for biological materials and food-processing systems, where inherent variability in raw materials often results in lower coefficients of determination. Similar ranges have been reported in previous optimization studies involving spray-dried food powders and functional protein ingredients, where the complexity of food matrices contributes to greater experimental variation. The close agreement between the R^2^ and adjusted R^2^ values for all responses further confirms that the developed models were not overfitted and that the included regression terms contributed meaningfully to explaining the observed variation. Likewise, the predicted R^2^ values generally agreed well with the adjusted R^2^ values, indicating satisfactory predictive performance across the experimental domain. Although the predicted R^2^ for wettability (0.59) was lower than its adjusted R^2^ (0.91), this response still exhibited an adequate signal-to-noise ratio and acceptable reproducibility, suggesting that wettability may be influenced by additional factors such as particle surface morphology, porosity, and surface composition that were not explicitly included in the model.

The coefficient of variation (CV), which measures experimental precision and reproducibility, remained below 5% for nearly all responses, indicating excellent repeatability of the experimental data. Slightly higher CV values were observed for wettability (7.13%) and foaming capacity (4.94%); however, these values remain acceptable for functional properties that are inherently sensitive to minor structural variations in protein-rich food systems. The relatively low CV values demonstrate that the experimental procedures and analytical measurements were both reliable and reproducible. Adequate precision, which evaluates the signal-to-noise ratio of the models, exceeded the recommended threshold value of 4 for every response, ranging from 11.86 for hygroscopicity to 32.88 for moisture content. Particularly high adequate precision values were obtained for powder yield, moisture content, whiteness, and emulsion activity index, indicating excellent model discrimination and confirming that these regression equations can be confidently used to navigate the design space and predict responses within the selected experimental region.

Collectively, these statistical indicators demonstrate that the response surface methodology successfully captured the relationships between spray-drying parameters and the quality attributes of aquafaba powder. The robustness of the models provides a reliable basis for process optimization and for predicting the effects of spray-drying conditions on both physical and techno-functional properties, thereby supporting the development of industrial-scale production strategies for functional aquafaba powder.

### 3.2. Effects of Spray-Drying Conditions on the Yield and Physical Properties of Aquafaba Powder

#### 3.2.1. Powder Yield (R1)

**Figure 1:**
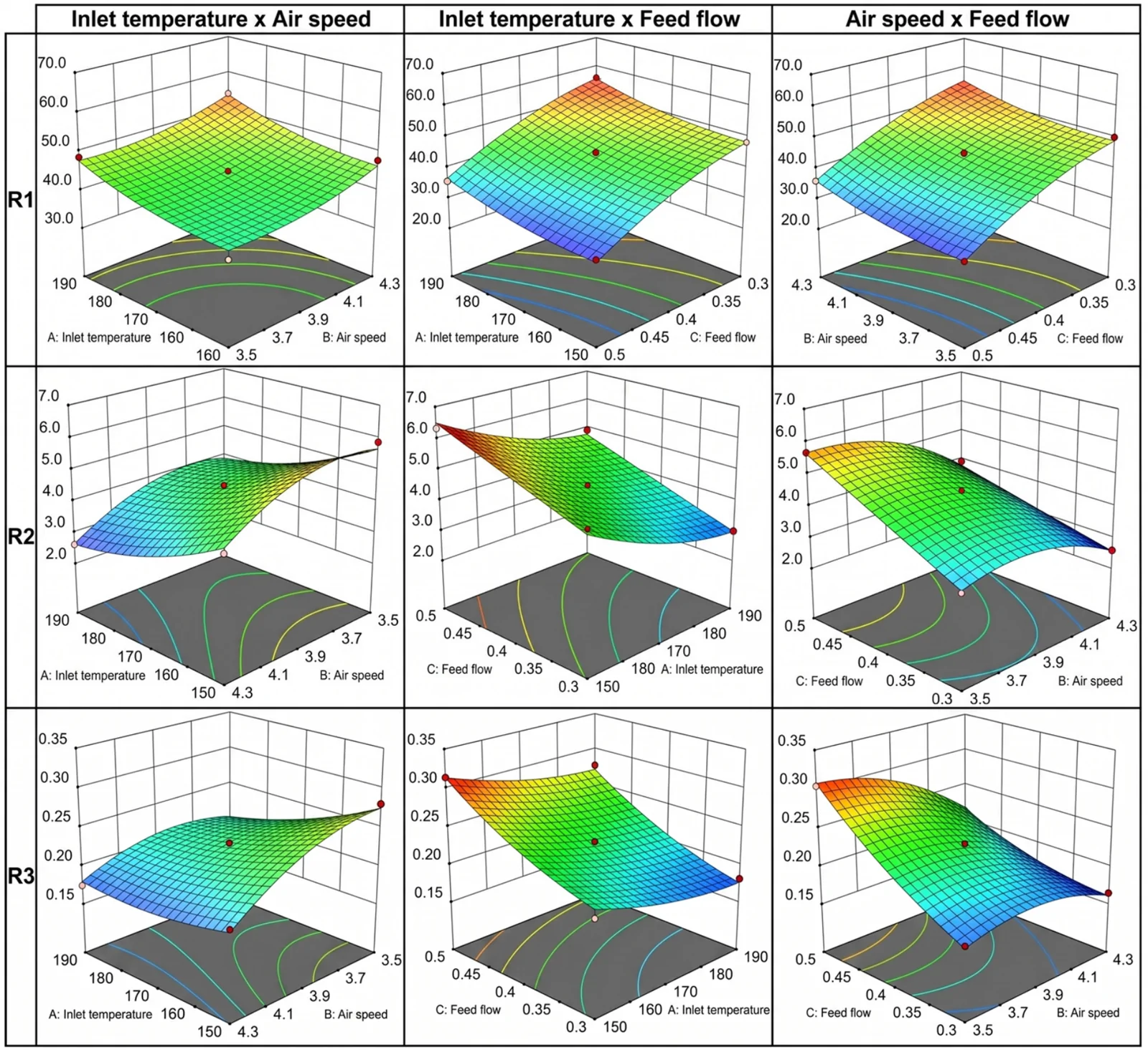
Three-dimensional graphs of process conditions interactions effect on powder yield (R1), moisture content (R2), and water activity (R3).

The powder yield of spray-dried aquafaba ranged from 30.03% to 55.95%, with the highest recovery obtained at an inlet air temperature of 190 °C, an air speed of 3.9 m/s, and a feed flow rate of 0.3 L/h (Table 1). Powder yield is one of the most important economic parameters in spray-drying because it directly influences process efficiency, product recovery, and overall manufacturing costs. Therefore, maximizing powder recovery while maintaining desirable functional properties is essential for the commercial production of aquafaba powder. Analysis of variance revealed that all three processing variables, inlet air temperature (A), air speed (B), and feed flow rate (C), as well as their quadratic terms (A^2^, B^2^, and C^2^) significantly influenced powder yield. Temperature and feed flow rate exhibited highly significant effects (*p* < 0.0001), whereas air speed also exerted a significant influence (*p* = 0.0003) (Table 3). The response surface plots (Figure 1) demonstrated that powder yield increased with increasing inlet air temperature and air speed but decreased with increasing feed flow rate. The positive effect of inlet air temperature on powder yield can be attributed to enhanced heat and mass transfer during drying. Higher drying temperatures increase the temperature gradient between the atomized droplets and the drying air, accelerating moisture evaporation and promoting the rapid formation of a dry outer crust on the particle surface. As a result, particle stickiness is reduced, minimizing adhesion to the drying chamber walls and improving powder recovery. Similar observations have been reported by Begliyev, Yavuz [9], who identified inlet air temperature as one of the most influential factors governing aquafaba powder yield. Comparable trends have also been widely reported for spray-dried dairy, fruit, and plant protein powders, in which elevated drying temperatures improved product recovery by reducing particle deposition in the drying chamber [6].

Air speed also contributed positively to powder recovery, although its effect was less pronounced than that of temperature. Higher air velocities enhance convective heat and mass transfer, facilitating more efficient moisture removal and shortening the residence time required to produce sufficiently dried particles. Consequently, powders with lower surface moisture exhibit reduced cohesiveness and are less likely to adhere to the chamber surfaces, thereby increasing collection efficiency. In contrast, increasing the feed flow rate negatively affected powder yield. A higher feed rate introduces a larger volume of liquid into the drying chamber, increasing droplet size and reducing the time available for complete moisture evaporation. Consequently, partially dried particles retain higher surface moisture and remain tacky, promoting adhesion to the drying chamber walls and cyclone separator, ultimately reducing powder recovery [6]. In addition, excessive feed rates may overwhelm the drying capacity of the system, further compromising drying efficiency and increasing material losses.

Overall, these findings demonstrate that inlet air temperature and feed flow rate are the dominant process variables governing aquafaba powder recovery, whereas air speed plays a complementary role by improving drying efficiency. The results further indicate that maximizing powder yield requires balancing rapid moisture removal with adequate residence time to minimize particle stickiness and wall deposition. Such optimization is particularly important for the commercial production of aquafaba powder, where improved product recovery directly translates into enhanced process efficiency, lower production costs, and greater industrial feasibility.

#### 3.2.2. Moisture Content (R2) and Water Activity (R3)

Moisture content and water activity are among the most critical quality attributes of spray-dried powders because they directly influence storage stability, microbial safety, caking behavior, and overall shelf life. Therefore, controlling these properties through process optimization is essential for producing high-quality aquafaba powder suitable for industrial applications.

The moisture content of the spray-dried aquafaba powders ranged from 2.59% to 6.30% (Table 1). The lowest moisture content was obtained at an inlet air temperature of 170 °C, an air speed of 4.3 m/s, and a feed flow rate of 0.3 L/h, whereas the highest value was recorded at 150 °C, 3.9 m/s, and 0.5 L/h. Analysis of variance demonstrated that inlet air temperature (A), air speed (B), and feed flow rate (C) all significantly affected moisture content (*p* < 0.0001). In addition, the interaction between air speed and feed flow rate (BC), together with the quadratic effects of temperature (A^2^) and air speed (B^2^), was statistically significant, indicating that moisture removal is governed by both the individual and combined effects of the processing variables (Table 3). As illustrated in Figure 1, moisture content decreased with increasing inlet air temperature and air speed but increased with increasing feed flow rate. The reduction in moisture content at higher drying temperatures is primarily attributed to enhanced heat and mass transfer. Increasing the inlet air temperature creates a greater temperature gradient between the atomized droplets and the drying air, accelerating water evaporation and shortening the drying time required to achieve low residual moisture. Likewise, higher air speeds improve convective heat transfer and facilitate moisture removal from the particle surface, resulting in more efficient drying. In contrast, increasing the feed flow rate introduces a greater liquid load into the drying chamber, reducing the residence time available for complete moisture evaporation and resulting in powders with higher residual moisture. These findings are consistent with those reported by Begliyev, Yavuz [9] and Atalar and Dervisoglu [6], who similarly observed that increasing drying temperature and airflow enhanced moisture removal efficiency during spray-drying of aquafaba and related food systems.

Water activity (a_w_) is an equally important quality parameter because it determines the availability of free water for microbial growth, enzymatic activity, and chemical deterioration. For most dried foods, a_w_ values between 0.20 and 0.40 are generally considered sufficiently low to inhibit microbial growth and substantially reduce the rates of deteriorative reactions. In the present study, the water activity of the aquafaba powders ranged from 0.16 to 0.31, indicating that all powders were within the range expected for microbiologically stable dried products. The lowest water activity was observed under the same conditions that yielded the lowest moisture content (170 °C, 4.3 m/s, and 0.3 L/h), demonstrating the effectiveness of these conditions for moisture removal.

All three primary processing variables significantly affected water activity, with feed flow rate exhibiting the strongest statistical influence (*p* < 0.0001). Significant interaction effects were also observed for AB and BC, together with the quadratic effects of B^2^ and C^2^ (Table 3), highlighting the complex relationship between drying conditions and the availability of unbound water within the powder matrix. Similar to the moisture content results, water activity decreased with increasing inlet air temperature and air speed and increased with higher feed flow rates. This close relationship between moisture content and water activity is expected because more efficient moisture removal reduces both the total water content and the proportion of free water available for microbial and chemical reactions.

These findings agree with those of Aslan and Ertaş [4], who reported that increasing the drying temperature significantly reduced the water activity of spray-dried aquafaba powder. Collectively, the results demonstrate that optimizing inlet air temperature, air speed, and feed flow rate effectively minimizes both moisture content and water activity, thereby improving storage stability, extending shelf life, and enhancing the commercial suitability of aquafaba powder for incorporation into dry food formulations.

#### 3.2.3. Powder Density (R4)

The powder density of spray-dried aquafaba ranged from 227.58 to 379.92 kg/m^3^, with the highest density obtained at an inlet air temperature of 170 °C, an air speed of 3.5 m/s, and a feed flow rate of 0.5 L/h (Table 1). Inlet air temperature (A), air speed (B), and feed flow rate (C) all significantly affected powder density (*p* = 0.0039, *p* = 0.0039, and *p* = 0.0008, respectively). In addition, the interactions between inlet temperature and air speed (AB), air speed and feed flow rate (BC), and the quadratic terms B^2^ and C^2^ were statistically significant (*p* < 0.05), demonstrating the combined influence of processing variables on particle formation (Table 3).

**Figure 2:**
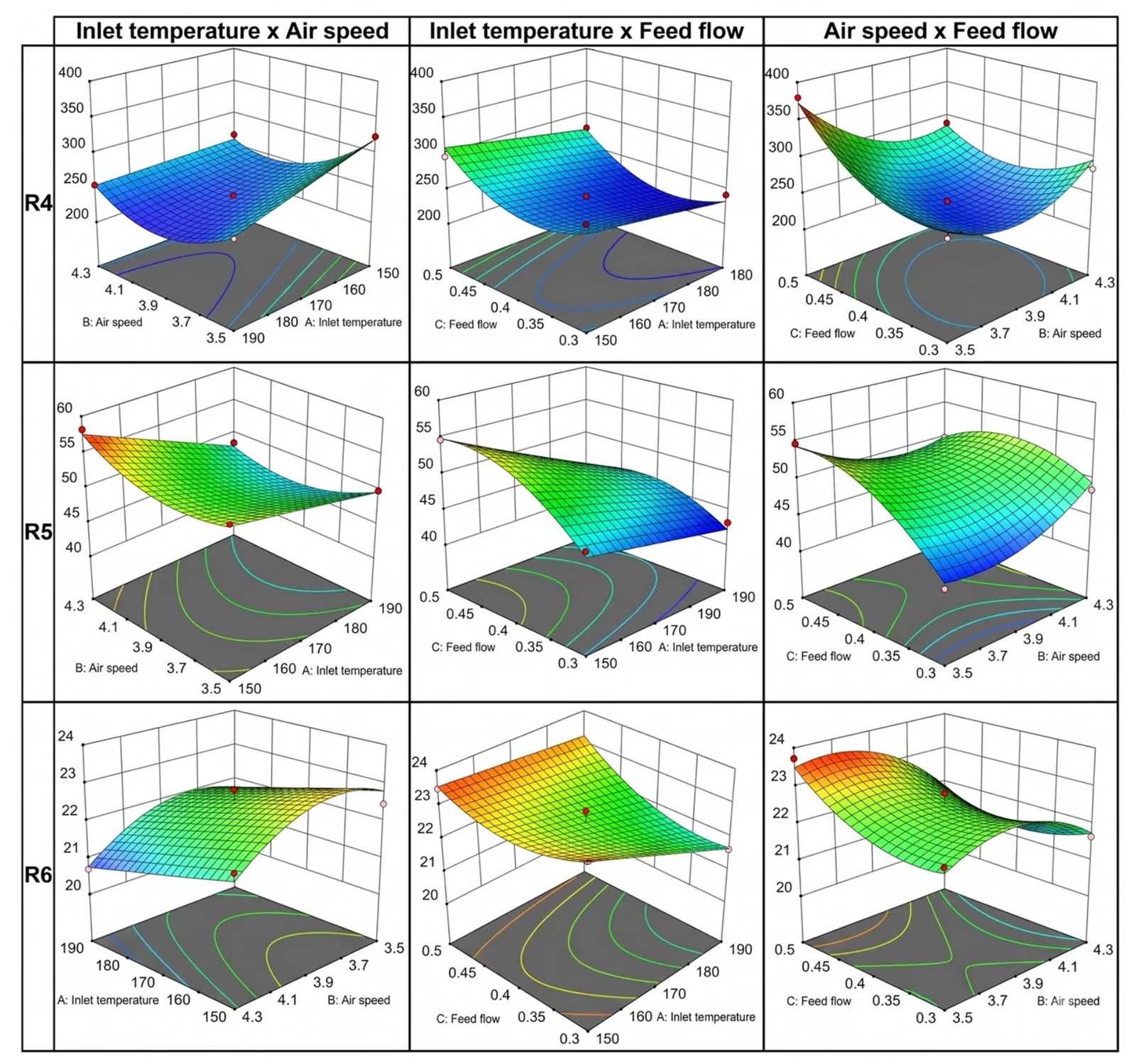
Three-dimensional graphs of process conditions interactions effect on powder density (R4), angle of repose (R5), and hygroscopicity (R6).

As shown in Figure 2, powder density decreased with increasing inlet air temperature and air speed but increased with higher feed flow rates. Higher drying temperatures promote rapid moisture evaporation, producing larger, more porous particles with lower bulk density, whereas higher feed flow rates increase the liquid load in the drying chamber, resulting in denser particles due to less efficient drying. Similar trends have been reported by Zouari, Mtibaa [18], who observed increased bulk density in spray-dried milk powders at higher feed rates, highlighting the important role of feed rate in determining particle structure and powder characteristics.

#### 3.2.4. Angle of Repose (R5)

The angle of repose (AOR), an indicator of powder flowability, ranged from 43.15° to 58.18°, with lower values indicating improved flow characteristics. Inlet air temperature (*p* < 0.0001) and feed flow rate (*p* = 0.0003) significantly influenced AOR, whereas air speed alone did not (*p* = 0.4067). However, the interaction between air speed and feed flow rate (*p* = 0.0027) and the quadratic effect of air speed (*p* = 0.0033) were significant (Table 3).

As illustrated in Figure 2, increasing the inlet air temperature reduced the angle of repose, indicating improved powder flowability. This behavior is likely associated with more efficient moisture removal, which reduces particle cohesiveness and inter-particle adhesion. In contrast, higher air speeds may generate finer particles that increase surface contact and friction, thereby reducing flowability. These findings agree with those of Tonon, Brabet [19], who reported improved flowability of spray-dried fruit powders at higher drying temperatures due to reduced stickiness and particle agglomeration [20]. Overall, optimizing drying temperature while maintaining a moderate air speed is important for producing aquafaba powder with desirable handling properties.

#### 3.2.5. Hygroscopicity (R6)

Hygroscopicity is an important indicator of powder stability because it reflects a material’s ability to absorb moisture from the surrounding environment during storage. The hygroscopicity of the aquafaba powders ranged from 20.70 to 23.72 g/100 g, with the lowest value obtained at 190 °C, 4.3 m/s, and 0.4 L/h, indicating reduced moisture uptake under more intensive drying conditions. All three processing variables significantly influenced hygroscopicity, including inlet air temperature (*p* = 0.0020), air speed (*p* = 0.0038), and feed flow rate (*p* = 0.0119) (Table 3). Significant effects were also observed for the interaction between temperature and feed flow rate (AC) and the quadratic terms B^2^ and C^2^. As shown in Figure 2, hygroscopicity decreased with increasing inlet air temperature and air speed but increased with higher feed flow rates. These results are consistent with previous studies on spray-dried acerola pomace extract [21], in which higher drying temperatures yielded powders with lower hygroscopicity. This behavior is likely due to the formation of more stable, glassy particle structures with reduced affinity for moisture, whereas insufficient drying at higher feed rates results in particles that readily absorb water during storage.

#### 3.2.6. Whiteness (R7)

The whiteness index ranged from 74.61 to 85.65, with the highest value obtained at 170 °C, 3.9 m/s, and 0.4 L/h, and the lowest at 150 °C, 3.9 m/s, and 0.5 L/h. Inlet air temperature and feed flow rate significantly affected whiteness (*p* < 0.0001), whereas air speed had no significant individual effect (*p* = 0.3258). However, significant BC interactions and quadratic effects (A^2^, B^2^, and C^2^) indicated a non-linear response to processing conditions (Table 3).

As illustrated in Figure 2, whiteness generally increased with increasing inlet air temperature until an optimum was reached, after which further increases in temperature may promote pigment degradation and non-enzymatic browning through Maillard reactions [22,23]. Similar reductions in color quality following thermal processing have been reported for chickpea protein concentrates [24]. In contrast, Koca, Erbay [25] found that drying temperature had little effect on the color of white cheese powder, emphasizing that the influence of spray-drying conditions on color depends on the composition of the food matrix. These results indicate that optimizing drying conditions is essential to preserve the visual quality of aquafaba powder while minimizing thermal discoloration.

### 3.3. Techno-Functional Properties

#### 3.3.1. Wettability (R8)

Wettability is a key indicator of the reconstitution behavior of food powders because it determines the rate at which water penetrates the powder particles during hydration. The shortest wetting time (158.00 s) was obtained at 190 °C, 3.9 m/s, and 0.5 L/h. Inlet air temperature (A, *p* = 0.0006), air speed (B, *p* = 0.0007), and feed flow rate (C, *p* = 0.0390) all significantly influenced wettability (Table 3). Although none of the interaction terms were significant, the quadratic effects of temperature (A^2^, *p* = 0.0003) and feed flow rate (C^2^, *p* = 0.0006) indicated non-linear responses to the drying conditions.

**Figure 3:**
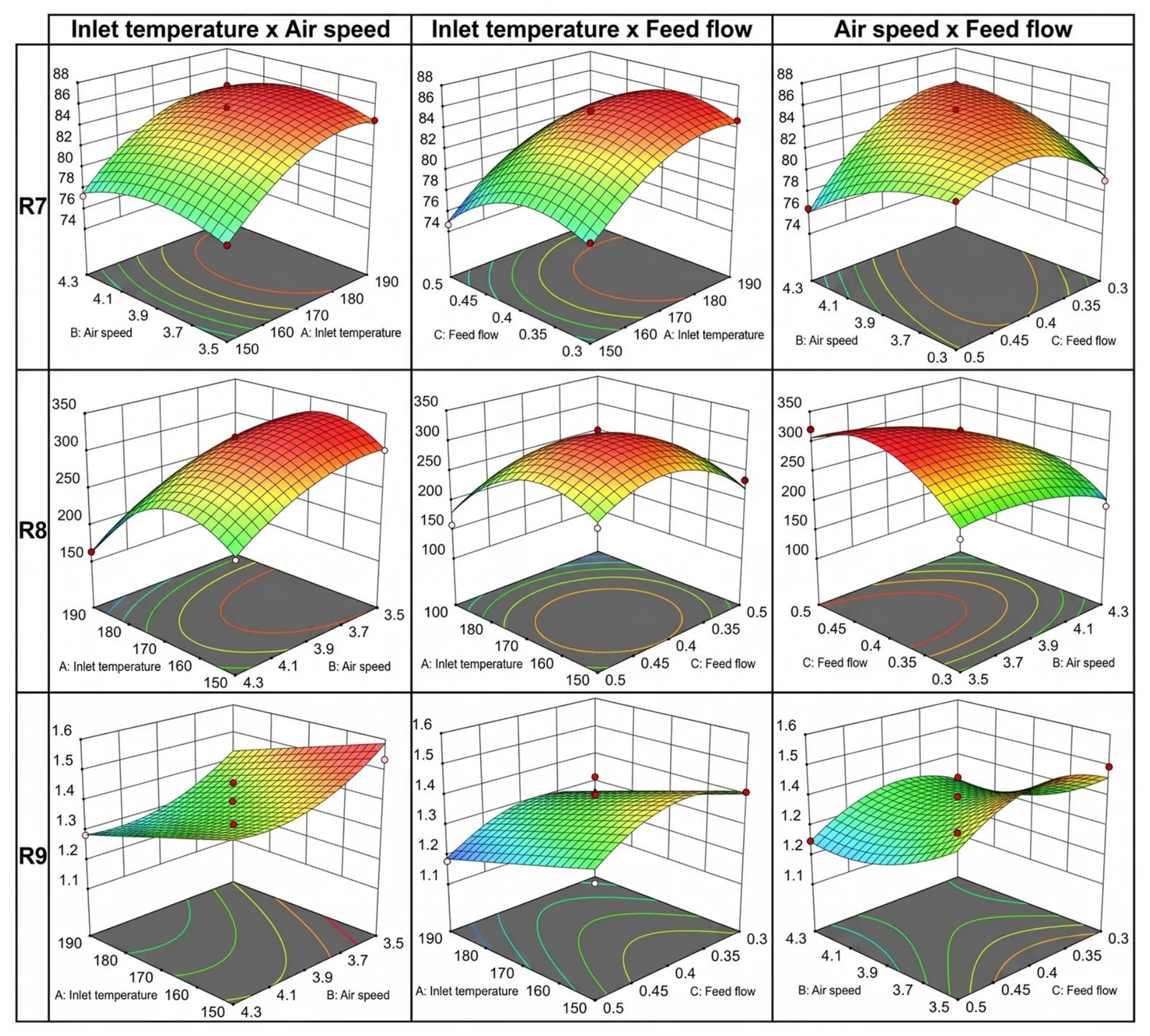
Three-dimensional graphs of process conditions interactions effect on whiteness (R7), wettability (R8), and water absorption capacity (R9).

As shown in Figure 3, wettability improved with increasing inlet air temperature and air speed but decreased at higher feed flow rates. Increased drying temperatures promote rapid moisture removal and reduce surface moisture, resulting in powders that hydrate more readily. However, excessively high temperatures may also produce a hard surface crust that limits water penetration into the particle core and slows subsequent dissolution [13]. These results demonstrate that an appropriate balance between drying intensity and particle structure is essential for achieving desirable reconstitution properties.

#### 3.3.2. Water (R9) and Oil (R11) Absorption Capacity

The water absorption capacity (WAC) of the aquafaba powders ranged from 1.18 to 1.53 g/g, with the highest value observed at 150 °C, 3.5 m/s, and 0.4 L/h. Inlet air temperature (*p* = 0.0026) and air speed (*p* = 0.0033) significantly affected WAC, whereas feed flow rate was not significant. However, the quadratic terms B^2^ (*p* = 0.0602) and C^2^ (*p* = 0.0132) indicated non-linear effects (Table 3). As illustrated in Figure 3, WAC decreased with increasing inlet air temperature and air speed, likely because rapid drying produced more compact particles with reduced porosity and fewer sites available for water retention. Similar trends have been reported by Padma, Rao [26] for spray-dried rice milk powder.

**Figure 4:**
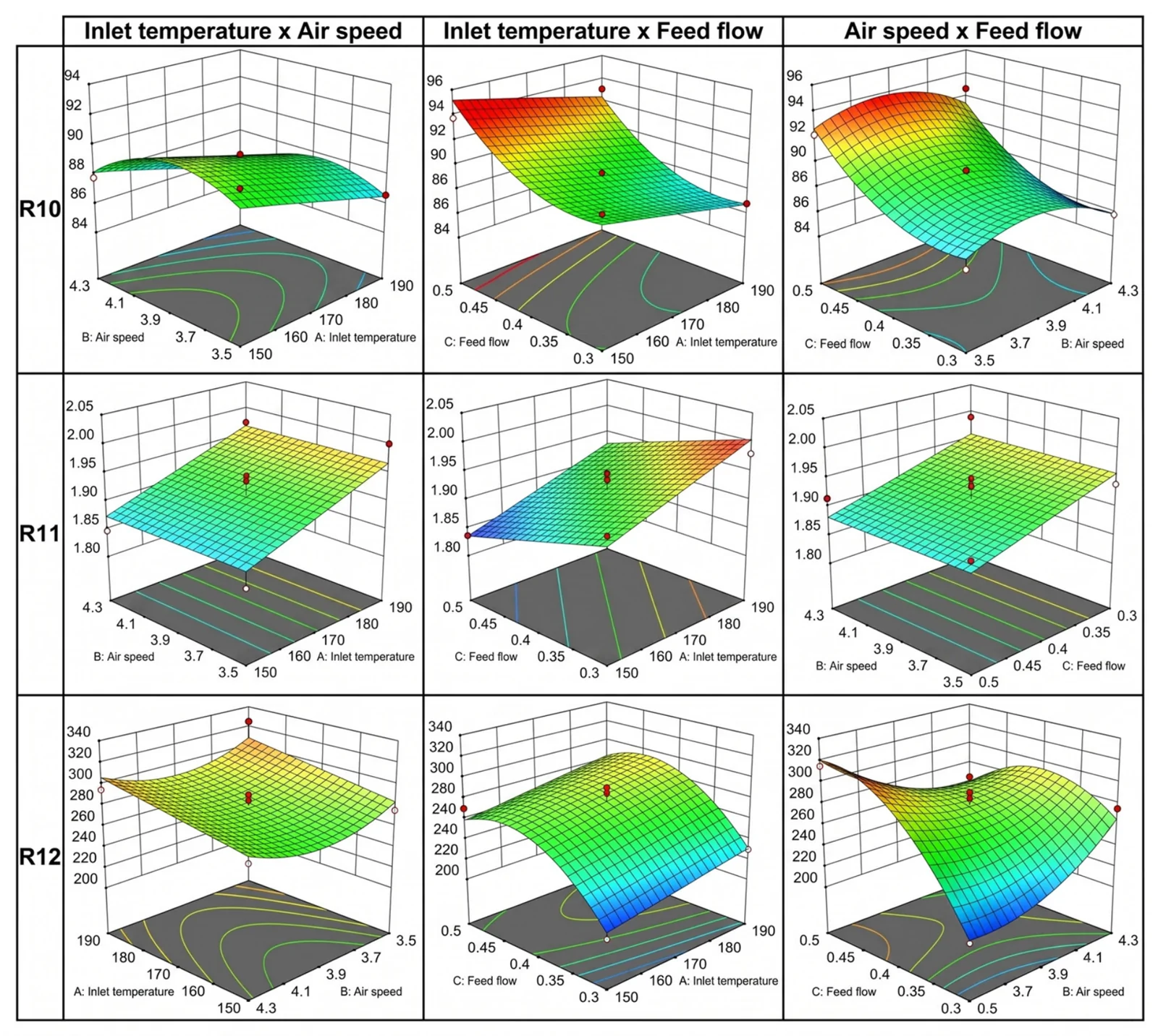
Three-dimensional graphs of process conditions interactions effect on water solubility index (R10), oil absorption capacity (R11), and foaming capacity (R12).

The oil absorption capacity (OAC) varied within a relatively narrow range of 1.84–2.00 g/g. Inlet air temperature (*p* = 0.0010) and feed flow rate (*p* = 0.0042) significantly affected OAC, whereas air speed and the interaction terms were not significant (Table 3). As shown in Figure 4, OAC decreased slightly as temperature and airspeed increased, likely due to the formation of denser particles with reduced porosity. These findings agree with Caliskan and Dirim [27], who reported reduced OAC in spray-dried powders produced at higher inlet temperatures. Because OAC is closely associated with particle porosity and surface hydrophobicity [28], moderate drying conditions appear to better preserve the structural characteristics required for oil retention.

#### 3.3.3. Water Solubility Index (R10)

The water solubility index (WSI) ranged from 84.60% to 93.75%, with the highest solubility obtained at 150 °C, 3.9 m/s, and 0.5 L/h (Table 1). Inlet air temperature (*p* = 0.0020) and feed flow rate (*p* < 0.0001) significantly influenced WSI, whereas air speed was not significant (*p* = 0.1059). Significant quadratic effects of B^2^ and C^2^ further indicated non-linear responses to the drying conditions (Table 3).

As illustrated in Figure 4, solubility generally increased with increasing inlet air temperature and moderate air speed. Higher drying temperatures likely promote more uniform particle formation and reduce surface stickiness, thereby enhancing powder dispersibility and dissolution. Similar improvements in solubility with increasing drying temperature have been reported for dairy and whey protein powders [29,30,31]. These findings indicate that appropriate drying conditions can improve the reconstitution properties of aquafaba powder without compromising its functional quality.

#### 3.3.4. Foaming Capacity (R12)

Foaming capacity (FC) ranged from 200.00% to 325.00%, demonstrating that spray-drying conditions strongly influenced the foaming properties of aquafaba powder (Table 1). Inlet air temperature (*p* = 0.0290) and feed flow rate (*p* = 0.0005) significantly affected FC, whereas air speed alone was not significant (*p* = 0.7972). However, the BC interaction (*p* = 0.0020) and the quadratic terms B^2^ (*p* = 0.0321) and C^2^ (*p* = 0.0016) were significant (Table 3).

As shown in Figure 4, higher foaming capacity was generally obtained at moderate to high inlet temperatures. This improvement is likely associated with partial protein denaturation, which exposes hydrophobic groups and enhances protein adsorption at the air–water interface, thereby promoting foam formation [32]. Similar improvements have been reported for spray-dried egg white powders [33]. In contrast, excessive thermal exposure may induce protein aggregation and reduce foaming performance, as reported by Franke and Kießling [34]. These results emphasize the importance of optimizing drying conditions to maximize foaming functionality while minimizing protein damage.

#### 3.3.5. Emulsion Activity Index (R13)

The emulsion activity index (EAI) ranged from 24.04 to 35.40 m^2^/g (Table 1). Although inlet air temperature did not have a significant individual effect (*p* = 0.0917), both air speed (*p* = 0.0002) and feed flow rate (*p* = 0.0021) significantly influenced EAI. Significant interaction and quadratic effects, including AB, AC, A^2^, and B^2^, further demonstrated the complex influence of spray-drying conditions on emulsifying properties (Table 3).

**Figure 5:**
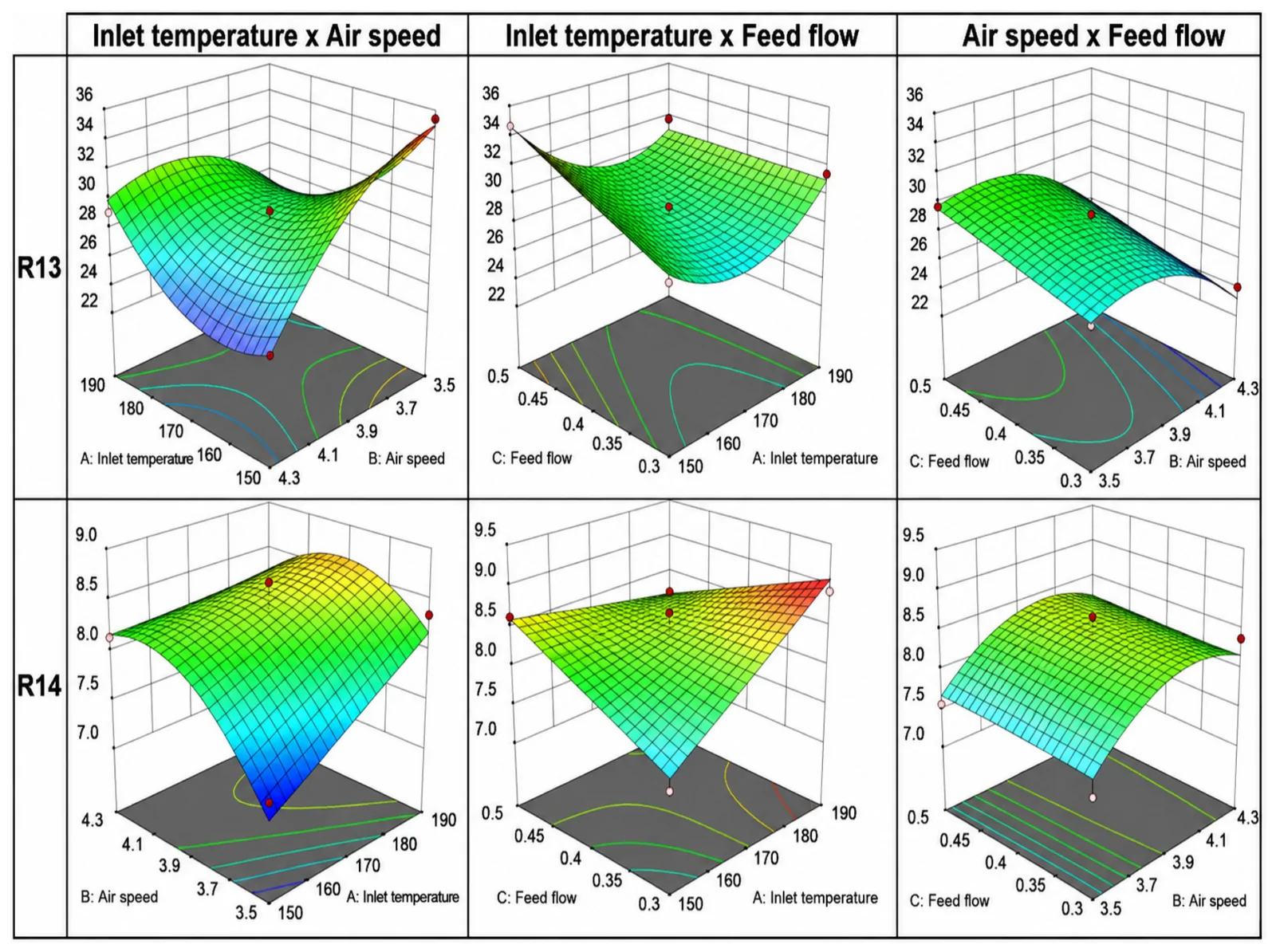
Three-dimensional graphs of process conditions interactions effect on emulsion activity index (R13), soluble protein content (R14).

As illustrated in Figure 5, EAI decreased with increasing inlet air temperature and air speed but increased with higher feed flow rates. Moderate drying conditions likely preserve protein solubility and maintain sufficient molecular flexibility for adsorption at the oil–water interface, thereby improving emulsifying performance [35,36]. Similar observations have been reported for chickpea-derived protein powders, in which preserving protein surface functionality was essential to maintaining emulsion stability [37].

#### 3.3.6. Soluble Protein Content (R14)

Soluble protein content is an important indicator of protein integrity and is closely associated with the emulsifying, foaming, and gelling properties of food ingredients. The soluble protein content of the aquafaba powders ranged from 7.29% to 8.93% (Table 1). Inlet air temperature (*p* = 0.0056) and air speed (*p* = 0.0070) significantly affected protein solubility, whereas feed flow rate was not significant (*p* = 0.6535). The interaction between temperature and feed flow rate (AC) was significant, while the remaining interaction terms were not (Table 3).

As shown in Figure 5, soluble protein content increased with increasing inlet air temperature and air speed and remained relatively unaffected by feed flow rate. Higher soluble protein levels indicate that the selected drying conditions preserved protein functionality without excessive denaturation. Similar trends have been reported for spray-dried milk proteins, where optimized drying temperatures maintained protein solubility while minimizing irreversible aggregation [38,39]. These findings highlight the importance of balancing drying efficiency with protein stability to maximize the techno-functional performance of aquafaba powder.

#### 3.3.7. Model Verification

To evaluate the adequacy and predictive performance of the optimized models, the experimental values for all responses were compared with their corresponding predicted values. The predicted and experimental results, together with their percentage differences, are presented in Table 4. The deviations between the predicted and observed values ranged from only 0.56% to 5.88%, demonstrating close agreement between the model estimates and the experimental measurements. These relatively small differences confirm that the developed models have strong predictive capability and can reliably describe the effects of the processing variables within the evaluated design space. Since all deviations were below 10%, the models can be considered sufficiently accurate and precise for optimizing aquafaba spray-drying conditions.

### 3.4. Comparison of Drying Methods

Spray drying (SD) and freeze drying (FD) are among the most widely used technologies for producing high-quality protein powders, each offering distinct advantages in terms of product quality, functionality, and processing efficiency. In this study, aquafaba powders produced by the two drying methods were compared for their physical, chemical, and techno-functional properties. It should be noted that freeze-dried samples required a gentle post-drying milling step to convert the lyophilized cake into a testable powder. In contrast, spray-dried samples yielded discrete particulates via atomization. Although mechanical milling inherently influences physical attributes such as particle size distribution, bulk density, and dissolution rate, this post-processing step represents the standard production pathway for freeze-dried functional ingredients. Consequently, the observed differences in functional and physical properties between the two drying regimes represent the cumulative effect of their respective complete processing lines.

The optimized SD process resulted in a significantly lower powder recovery (57.59%) than FD (99.85%). This difference is primarily attributed to the laboratory-scale spray dryer, in which adhesion of fine particles to the drying chamber walls and cyclone losses commonly reduce powder recovery compared with freeze-drying. Despite the lower recovery, SD powders exhibited significantly lower moisture content and water activity than FD powders, demonstrating that the optimized spray-drying conditions produced powders with superior storage stability through more efficient moisture removal.

The physical properties also differed considerably between the two drying methods. The density of SD aquafaba (256.08 kg/m^3^) was substantially lower than that of FD aquafaba (655.58 kg/m^3^), reflecting differences in particle formation during drying. SD powders also exhibited a higher angle of repose (44.9°) and greater hygroscopicity (21.21%) than FD powders, indicating reduced flowability and increased moisture uptake. These characteristics are likely related to the smaller particle size and greater inter-particle interactions of spray-dried powders. Conversely, SD powders exhibited a substantially higher water solubility index (88.44%), whereas FD powders wetted much more rapidly (30.33 s) than SD powders (136.17 s). These results demonstrate that although freeze drying facilitates rapid water penetration, spray drying produces powders with superior dissolution characteristics.

The optimized SD process also effectively preserved and, in some cases, enhanced the functional properties of aquafaba proteins. Spray-dried aquafaba exhibited higher foaming capacity (266.67%) and greater soluble protein content (8.6%) than FD powder, suggesting that the selected drying conditions maintained the protein functionality required for foam formation. In addition, SD powder showed a significantly higher oil absorption capacity but a lower water absorption capacity than FD powder (*p* < 0.05). The reduced water absorption is consistent with observations reported for other pulse protein powders, including lentil protein isolates. Notably, no significant differences were observed between SD and FD powders in emulsion activity, indicating that both drying methods adequately preserved the emulsifying functionality of aquafaba proteins.

**Figure 6:**
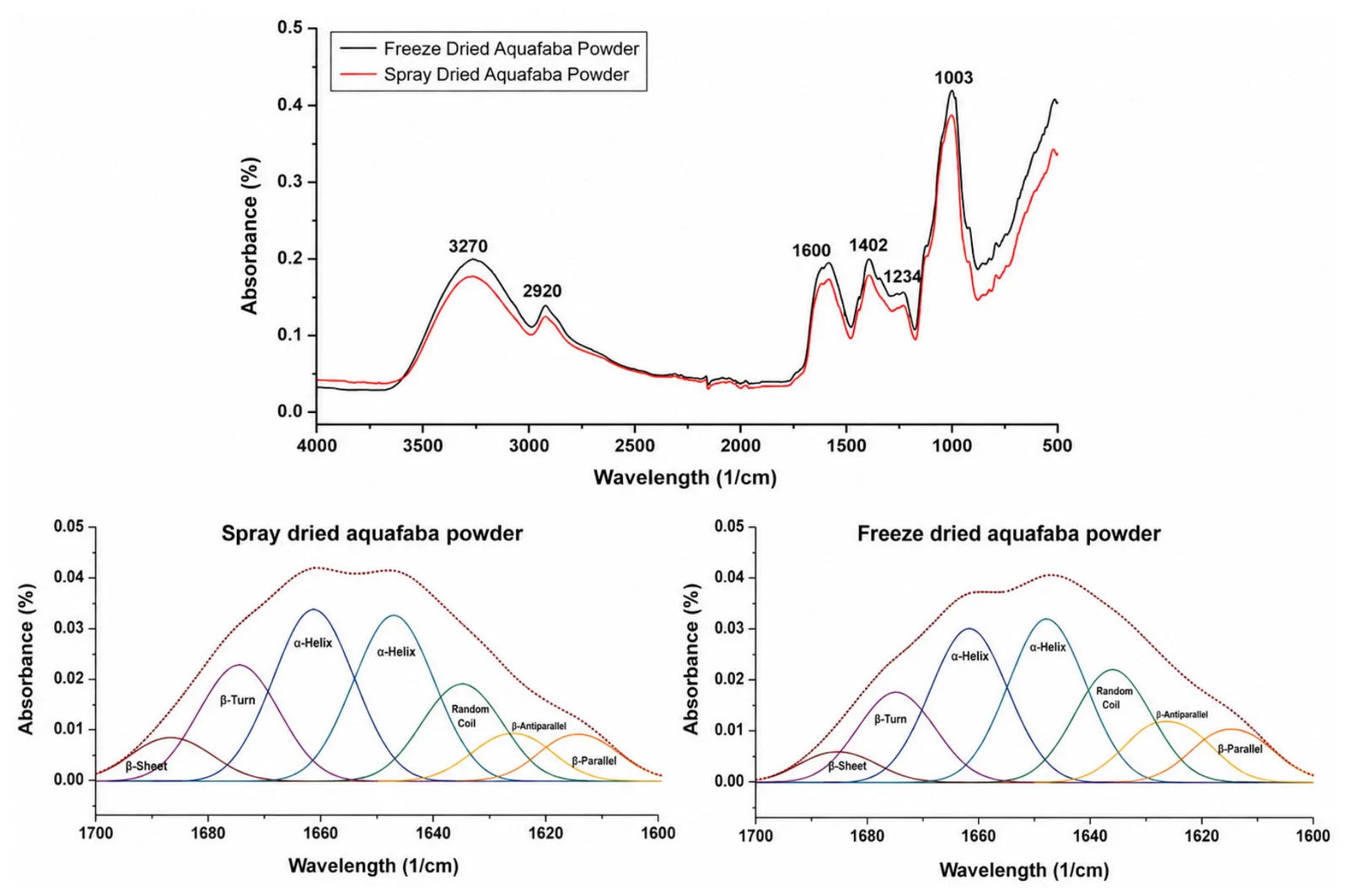
FTIR spectra and secondary structure graphs of spray-dried and freeze-dried aquafaba powder.

FTIR and protein secondary structure: FTIR spectroscopy was used to evaluate the effect of drying method on the chemical structure and functional groups of aquafaba powders. As shown in Figure 6, both FD and optimized SD powders exhibited nearly identical FTIR spectra, indicating that the fundamental chemical composition of aquafaba remained largely unchanged despite exposure to the elevated temperatures associated with spray drying. The broad absorption band at 3270 cm^−1^ corresponds to O–H and N–H stretching vibrations associated with hydroxyl groups in carbohydrates and amide groups in proteins. The peak at 2920 cm^−1^ represents asymmetric stretching of C–H bonds from aliphatic protein and lipid chains. The prominent Amide I band near 1600 cm^−1^, which primarily reflects C=O stretching vibrations, remained well preserved after spray drying, suggesting that the overall secondary protein structure was maintained. This observation is consistent with the excellent foaming and emulsifying properties observed for the SD powders.

Additional characteristic peaks at 1402 cm^−1^ and 1234 cm^−1^ correspond to C–H bending vibrations and combined C–N stretching/N–H bending vibrations, respectively, further confirming preservation of the protein matrix. The peak at 1003 cm^−1^ represents C–O and C–C stretching vibrations within polysaccharides, indicating that the carbohydrate fraction of aquafaba also remained structurally intact after spray drying. Although the overall absorbance intensity was slightly higher for FD powders, likely because of their greater porosity and differences in particle packing during FTIR measurements, no new absorption bands or significant peak shifts were observed. These findings demonstrate that optimized spray drying does not induce detrimental chemical modifications or degradation of the major protein and carbohydrate components of aquafaba.

To further investigate structural changes, the Amide I region (1600–1700 cm^−1^) was deconvoluted to quantify protein secondary structures ($R^{2}>0.99$, Gaussian fitting error). The α-helix content remained essentially unchanged, increasing only slightly from 48.98% (FD) to 49.12% (SD). This minimal difference indicates that spray drying at 189 °C preserved the core helical regions of aquafaba proteins, consistent with the comparable emulsion activity observed between the two drying methods. In contrast, SD powders exhibited increases in β-sheet (19.10% to 20.09%) and β-turn (13.70% to 16.79%) structures, accompanied by a decrease in random coil content from 18.21% to 13.99%. These changes suggest a controlled thermal rearrangement toward a more ordered protein conformation rather than extensive denaturation.

The reduction in random coil structures likely reflects partial protein unfolding, which exposes hydrophobic regions that facilitate adsorption at the air–water interface [32]. Such molecular rearrangements indicate a plausible explanation for the significantly higher foaming capacity (266.67%) observed in SD powder. Overall, the FTIR and secondary structure analyses suggest that optimized spray drying preserves the molecular integrity of aquafaba proteins while inducing subtle conformational changes that enhance specific techno-functional properties, particularly foaming performance, without compromising emulsifying functionality. While a minor shift toward folded or ordered secondary conformers is often reported to accompany protein structural re-arrangements, this marginal change alone cannot be definitively established as the primary causal driver of the improved foaming performance. Rather, the enhanced techno-functional properties are more plausibly governed by a synergistic combination of interfacial tension reduction, particle solubility, surface charge, and particle size distribution resulting from the drying conditions, with secondary structure changes providing supportive structural context rather than direct causation.

**Figure 7:**
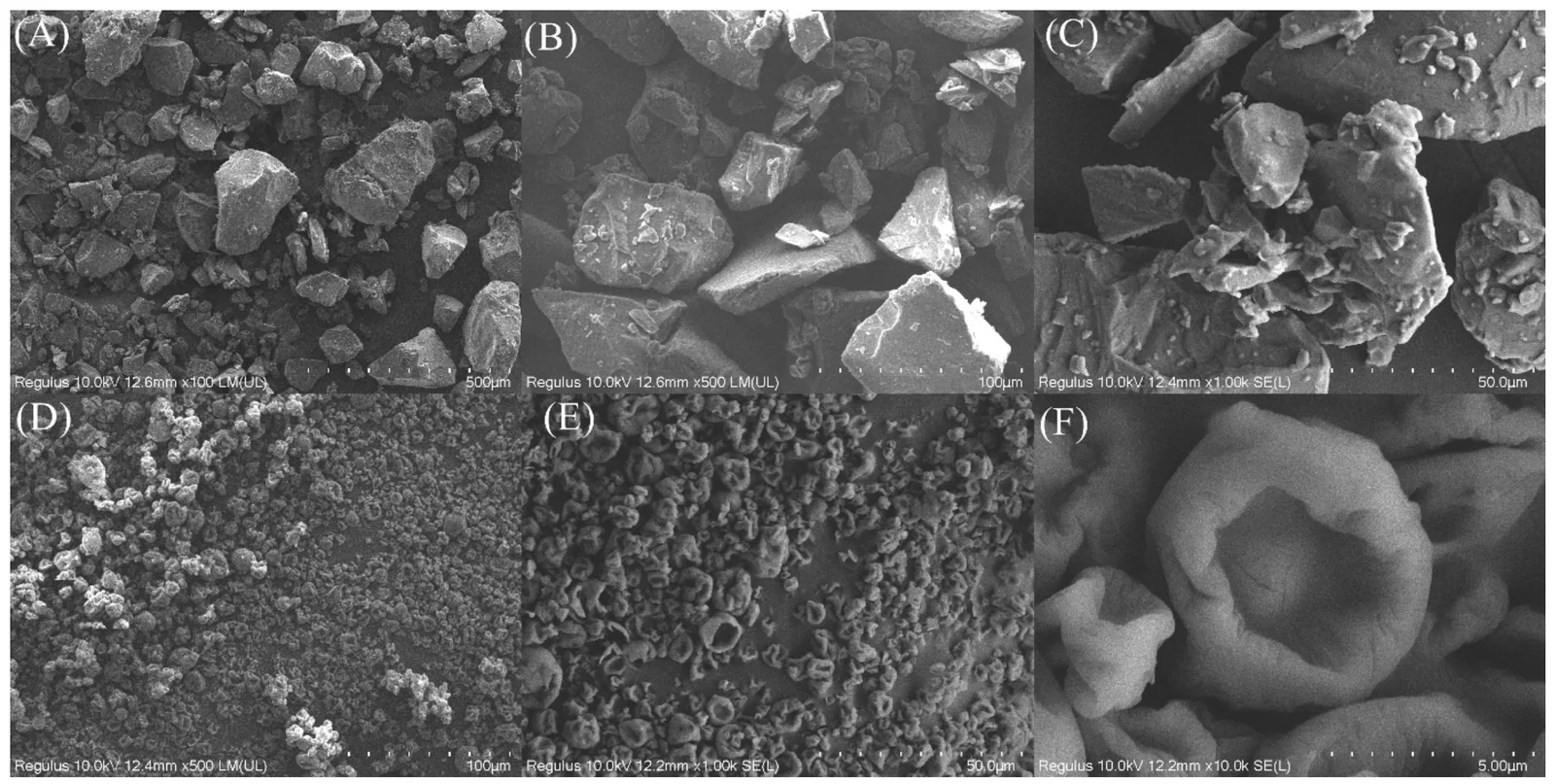
SEM images of freeze-dried (**A**–**C**) and spray-dried (**D**–**F**) aquafaba powders.

Representative SEM micrographs (Figure 7) further illustrate the distinct particle morphologies generated by the two drying methods. Freeze-dried aquafaba consisted of large, irregular, sharp-edged flakes with a glass-like appearance, which is characteristic of freeze-dried materials. During freeze-drying, sublimation of ice crystals leaves behind a rigid, highly porous matrix while preserving the original frozen structure, as moisture is removed without surface-tension effects. Although this porous network facilitates rapid water penetration, the irregular particle geometry contributes to poor flowability.

The highly porous, flake-like structure of FD powder explains its markedly faster wettability (30.33 s) compared with that of denser spray-dried particles. In contrast, spray-dried aquafaba (Figure 7D–F) consisted of much smaller, discrete spherical particles exhibiting varying degrees of surface indentation. At higher magnification, the particles displayed the characteristic shriveled or collapsed morphology commonly observed in spray-dried materials. This morphology develops when rapid moisture evaporation forms a rigid outer shell, which subsequently collapses as internal vapor pressure decreases during the final stages of drying.

The generally spherical geometry of SD particles favors powder handling compared with the angular FD flakes. However, their smaller size and wrinkled surfaces increase surface area and inter-particle friction, contributing to the higher angle of repose (44.9°) measured for SD powder. At the same time, the smaller and more uniform particles exhibited a higher water solubility index (88.44%) and greater light scattering, resulting in a significantly higher whiteness index (82.37) than the larger FD flakes. Collectively, the SEM observations complement the physical and functional analyses, demonstrating that spray drying produces structurally distinct particles that contribute to improved solubility and functional performance while maintaining the chemical integrity of aquafaba proteins.

## 4. Conclusions

This study successfully optimized the spray-drying process for producing high-quality aquafaba powder using Response Surface Methodology (RSM). The results demonstrated that inlet air temperature, air speed, and feed flow rate significantly influenced the physical, chemical, and techno-functional properties of the powder through both individual and interactive effects. The optimal processing conditions were identified as an inlet air temperature of 189 °C, an air speed of 4.2 m/s, and a feed flow rate of 0.3 L/h. Under these conditions, the developed models exhibited high predictive accuracy, with excellent agreement between the predicted and experimental values, confirming the robustness of the optimization approach.

A comparison of the optimized spray-dried powder with freeze-dried aquafaba demonstrated that spray drying offers several important advantages while preserving the functional integrity of aquafaba proteins. Spray-dried powder exhibited significantly lower moisture content and water activity, indicating improved storage stability, as well as higher whiteness, greater water solubility, enhanced oil absorption capacity, and superior foaming performance. Although freeze-dried aquafaba exhibited faster wettability and greater water absorption capacity, spray drying effectively preserved the protein structure, as confirmed by FTIR analysis, while promoting favorable conformational changes that improved techno-functional performance without compromising emulsifying properties.

Beyond statistical optimization, these findings highlight the practical feasibility of transforming a low-value legume processing side stream into a standardized, shelf-stable functional food ingredient. Industrially, the optimized spray-drying process provides a scalable, continuous, and efficient manufacturing framework that overcomes the rapid spoilage and batch variability of liquid aquafaba. Due to its preserved solubility, foaming, and emulsifying properties, the resulting aquafaba powder holds strong commercial potential as a clean-label, plant-based egg-white replacer in various food formulations, including egg-free mayonnaise, bakery products, confectioneries, and aerated desserts.

While this study successfully establishes optimized processing conditions for bench-scale aquafaba spray drying, certain methodological limitations should be noted. First, although functional retentions were systematically mapped, the underlying molecular structure-function relationships across all multivariable conditions warrant deeper mechanistic investigation. Second, the practical industrial viability of the process was evaluated at a laboratory scale. Consequently, future research must focus on pilot-scale upscaling, detailed energy consumption analysis, techno-economic feasibility assessment, and product application trials to fully validate its commercial viability.

## Figures and Tables

**Table 1:** Box–Behnken experimental design matrix for the spray drying process and measured data for each response parameter.

Run	A	B	C	R1	R2	R3	R4	R5	R6	R7	R8	R9	R10	R11	R12	R13	R14
1	150.0	3.5	0.4	38.06 ± 0.43	5.87 ± 0.21	0.281 ± 0.012	323.70 ± 3.87	54.65 ± 2.87	22.45 ± 0.04	78.08 ± 0.08	301.50 ± 9.19	1.53 ± 0.02	90.51 ± 0.83	1.85 ± 0.01	275.00 ± 7.07	35.4 ± 1.66	7.29 ± 0.25
2	190.0	3.5	0.4	48.65 ± 0.64	3.74 ± 0.11	0.198 ± 0.005	256.09 ± 2.68	49.89 ± 2.52	21.70 ± 0.42	84.51 ± 0.36	236.00 ± 5.66	1.40 ± 0.02	86.61 ± 2.52	2.00 ± 0.01	325.00 ± 7.07	27.1 ± 1.25	8.35 ± 0.44
3	150.0	4.3	0.4	47.70 ± 0.28	4.22 ± 0.16	0.199 ± 0.006	265.85 ± 2.62	58.18 ± 3.24	22.06 ± 0.30	77.23 ± 0.07	229.50 ± 4.95	1.49 ± 0.03	87.76 ± 0.44	1.85 ± 0.03	275.00 ± 7.07	24.8 ± 0.27	8.13 ± 0.31
4	190.0	4.3	0.4	53.82 ± 0.85	2.63 ± 0.16	0.174 ± 0.008	254.43 ± 2.56	48.43 ± 2.52	20.70 ± 0.84	84.18 ± 0.35	163.50 ± 6.36	1.29 ± 0.01	84.60 ± 1.44	1.97 ± 0.00	295.00 ± 7.07	29.2 ± 2.72	8.22 ± 0.49
5	150.0	3.9	0.3	48.21 ± 0.49	4.85 ± 0.04	0.208 ± 0.005	276.21 ± 0.59	46.97 ± 0.00	23.55 ± 0.04	78.48 ± 0.17	235.00 ± 5.66	1.41 ± 0.01	90.37 ± 0.54	1.93 ± 0.02	200.00 ± 0.00	28.9 ± 0.95	7.42 ± 0.04
6	190.0	3.9	0.3	55.95 ± 0.71	2.98 ± 0.23	0.181 ± 0.011	241.63 ± 2.88	43.15 ± 0.00	21.64 ± 0.36	84.71 ± 0.24	165.50 ± 6.36	1.25 ± 0.04	86.82 ± 0.64	1.98 ± 0.00	230.00 ± 14.14	31.5 ± 2.04	8.93 ± 0.09
7	150.0	3.9	0.5	30.27 ± 0.35	6.30 ± 0.16	0.314 ± 0.008	295.43 ± 2.02	54.65 ± 2.87	23.47 ± 0.75	74.61 ± 0.07	243.50 ± 2.12	1.30 ± 0.02	93.75 ± 1.44	1.84 ± 0.01	270.00 ± 0.00	34.7 ± 0.14	8.61 ± 0.11
8	190.0	3.9	0.5	35.56 ± 0.56	4.92 ± 0.11	0.277 ± 0.010	277.72 ± 4.24	44.43 ± 2.21	23.11 ± 0.03	81.02 ± 0.05	158.00 ± 5.66	1.18 ± 0.02	93.10 ± 0.88	1.88 ± 0.01	270.00 ± 14.14	31.7 ± 0.95	8.22 ± 0.20
9	170.0	3.5	0.3	50.19 ± 0.78	3.24 ± 0.21	0.178 ± 0.011	266.40 ± 6.80	43.15 ± 0.00	22.25 ± 0.39	79.08 ± 0.33	226.50 ± 3.54	1.50 ± 0.02	86.26 ± 0.18	1.94 ± 0.01	205.00 ± 7.07	26.8 ± 0.03	7.40 ± 0.29
10	170.0	4.3	0.3	54.22 ± 0.92	2.59 ± 0.17	0.165 ± 0.001	284.62 ± 0.43	48.43 ± 2.52	21.64 ± 0.26	84.31 ± 0.59	191.50 ± 3.54	1.27 ± 0.01	85.74 ± 0.89	1.99 ± 0.00	275.00 ± 7.07	24.0 ± 0.17	8.39 ± 0.28
11	170.0	3.5	0.5	30.03 ± 0.21	5.67 ± 0.25	0.305 ± 0.009	379.92 ± 6.68	54.65 ± 2.87	23.72 ± 0.81	82.12 ± 0.17	320.50 ± 3.54	1.45 ± 0.01	92.15 ± 1.31	1.90 ± 0.01	315.00 ± 7.07	29.7 ± 0.58	7.55 ± 0.35
12	170.0	4.3	0.5	36.00 ± 0.43	3.93 ± 0.12	0.214 ± 0.004	289.68 ± 2.93	49.89 ± 2.52	21.85 ± 0.26	76.44 ± 0.58	220.00 ± 8.49	1.25 ± 0.01	92.69 ± 1.36	1.91 ± 0.01	265.00 ± 7.07	25.7 ± 0.10	8.03 ± 0.69
13	170.0	3.9	0.4	41.86 ± 0.64	4.29 ± 0.11	0.221 ± 0.007	239.49 ± 1.33	48.43 ± 2.52	22.03 ± 0.00	84.60 ± 0.18	319.00 ± 7.07	1.46 ± 0.04	89.37 ± 0.67	1.94 ± 0.01	285.00 ± 7.07	28.5 ± 0.24	8.31 ± 0.06
14	170.0	3.9	0.4	44.98 ± 0.28	4.31 ± 0.15	0.231 ± 0.005	227.58 ± 1.09	49.89 ± 2.52	22.84 ± 0.35	84.35 ± 0.31	305.50 ± 3.54	1.32 ± 0.05	88.93 ± 0.82	1.88 ± 0.02	290.00 ± 0.00	27.6 ± 0.03	8.33 ± 0.17
15	170.0	3.9	0.4	42.84 ± 0.71	4.52 ± 0.19	0.221 ± 0.004	231.10 ± 0.59	49.89 ± 2.52	22.79 ± 0.48	85.65 ± 0.28	312.00 ± 4.24	1.40 ± 0.05	89.04 ± 0.42	1.95 ± 0.02	265.00 ± 7.07	29.2 ± 0.27	8.67 ± 0.27

A (inlet air temperature), B (air flow rate), C (feed flow rate). R1 (powder yield), R2 (moisture content), R3 (water activity), R4 (powder density), R5 (angle of repose), R6 (hygroscopicity), R7 (whiteness), R8 (wettability), R9 (water absorption capacity), R10 (water solubility index), R11 (oil absorption capacity), R12 (foaming capacity), R13 (emulsion activity index), R14 (soluble protein content).

**Table 2:** Model fitting values for each response parameter.

Response	Min	Max	Mean	SD	C.V.	R^2^	Ad. R^2^	Pr. R^2^	Ad. Prec.
R1	30.03	55.95	43.89	1.46	3.33	0.98	0.97	0.94	26.67
R2	2.59	6.30	4.27	0.18	4.10	0.99	0.98	0.95	32.88
R3	0.16	0.31	0.22	0.01	3.73	0.99	0.97	0.89	23.55
R4	227.58	379.92	273.99	10.99	4.01	0.96	0.92	0.76	17.49
R5	43.15	58.18	49.65	1.02	2.06	0.98	0.95	0.83	19.34
R6	20.70	23.72	22.39	0.32	1.44	0.93	0.86	0.75	11.86
R7	74.61	85.65	81.29	0.55	0.67	0.99	0.98	0.93	24.88
R8	158.00	320.50	241.83	17.24	7.13	0.96	0.91	0.59	12.94
R9	1.18	1.53	1.36	0.05	3.84	0.85	0.77	0.61	12.10
R10	84.60	93.75	89.18	0.93	1.04	0.93	0.90	0.79	16.77
R11	1.84	2.00	1.92	0.03	1.60	0.72	0.67	0.54	12.38
R12	200.00	325.00	269.33	13.31	4.94	0.92	0.86	0.69	12.55
R13	24.04	35.40	28.99	0.78	2.69	0.97	0.94	0.82	20.60
R14	7.29	8.93	8.12	0.21	2.63	0.89	0.81	0.62	13.41

R1 (powder yield), R2 (moisture content), R3 (water activity), R4 (powder density), R5 (angle of repose), R6 (hygroscopicity), R7 (whiteness), R8 (wettability), R9 (water absorption capacity), R10 (water solubility index), R11 (oil absorption capacity), R12 (foaming capacity), R13 (emulsion activity index), R14 (soluble protein content).

**Table 3:** ANOVA table showing the variables as linear, quadratic, and interaction terms for each response in spray-dried aquafaba powders.

	Model	A	B	C	AB	AC	BC	A^2^	B^2^	C^2^	LoF
R1	<0.0001	<0.0001	0.0003	<0.0001				0.0402	0.0319	0.0093	0.6539
R2	<0.0001	<0.0001	<0.0001	<0.0001			0.0138	0.0082	0.0002		0.3392
R3	<0.0001	0.0004	0.0001	<0.0001	0.0134		0.0036	0.0799	0.0032	0.0380	0.2660
R4	0.0002	0.0039	0.0039	0.0008	0.0378		0.0017		0.0003	0.0005	0.2069
R5	0.0002	<0.0001	0.4067	0.0003	0.0509	0.0204	0.0027		0.0033	0.0013	0.3984
R6	0.0016	0.0020	0.0038	0.0119		0.0473	0.0926		0.0045	0.0167	0.8789
R7	<0.0001	<0.0001	0.3258	<0.0001			<0.0001	<0.0001	0.0010	<0.0001	0.7792
R8	0.0003	0.0006	0.0007	0.0390			0.0992	0.0003	0.0590	0.0006	0.1060
R9	0.0017	0.0026	0.0033	0.1157					0.0602	0.0132	0.8308
R10	<0.0001	0.0020	0.1059	<0.0001					0.0051	0.0038	0.0465
R11	0.0005	0.0010		0.0042							0.6848
R12	0.0006	0.0290	0.7972	0.0005			0.0020		0.0321	0.0016	0.5730
R13	<0.0001	0.0917	0.0002	0.0021	< 0.0001	0.0094		0.0002	0.0008		0.5722
R14	0.0016	0.0056	0.0070	0.6535	0.0514	0.0022			0.0043		0.5361

A (inlet air temperature), B (air flow rate), C (feed flow rate). R1 (powder yield), R2 (moisture content), R3 (water activity), R4 (powder density), R5 (angle of repose), R6 (hygroscopicity), R7 (whiteness), R8 (wettability), R9 (water absorption capacity), R10 (water solubility index), R11 (oil absorption capacity), R12 (foaming capacity), R13 (emulsion activiy index), R14 (soluble protein content).

**Table 4:** Experimental validation data of predicted values at optimal conditions and comparation with freeze dried powder.

Parameters	Goal	Predicted Results	Experimental Results	Difference (%)	Freeze-Dried Aquafaba
Yield	maximize	57.95	57.59 ± 0.46 ^a^	0.62	99.85 ± 0.05 ^b^
Moisture content	minimize	2.52	2.47 ± 0.13 ^a^	1.98	3.51 ± 0.35 ^b^
Water activity	minimize	0.17	0.18 ± 0.01 ^a^	5.88	0.34 ± 0.01 ^b^
Density	maximize	262.13	256.08 ± 10.87 ^a^	2.31	655.58 ± 10.06 ^b^
Angle of repose	minimize	44.65	44.9 ± 2.56 ^a^	0.56	37.56 ± 1.43 ^b^
Hygroscopicity	minimize	21.33	21.21 ± 0.29 ^a^	0.56	18.18 ± 0.11 ^b^
Whiteness	maximize	85.65	82.37 ± 0.3 ^a^	3.83	65.52 ± 2 ^b^
Wettability	minimize	143.29	136.17 ± 2.47 ^a^	4.97	30.33 ± 2.84 ^b^
Water absorption capacity	maximize	1.24	1.21 ± 0.02 ^a^	2.42	2.63 ± 0.06 ^b^
Water solubility index	maximize	85.73	88.44 ± 0.84 ^a^	3.16	74.47 ± 1.57 ^b^
Oil absorption capacity	maximize	2.00	2.03 ± 0.08 ^a^	1.50	1.93 ± 0.04 ^a^
Foaming capacity	maximize	258.39	266.67 ± 2.89 ^a^	3.20	243.33 ± 2.89 ^b^
Emulsion activity index	maximize	30.33	29.76 ± 0.17 ^a^	1.88	29.54 ± 0.42 ^a^
Soluble protein content	maximize	8.86	8.6 ± 0.26 ^a^	2.93	8.02 ± 0.18 ^b^

^a,b^ Mean values within the same row carrying different superscripts differ significantly (*p* < 0.05). Results are presented as mean ± SD (n = 3).

## Data Availability

The data supporting the findings of this study are available from the corresponding authors upon reasonable request.
